# Potential influence of socioeconomic status on genetic correlations between alcohol consumption measures and mental health

**DOI:** 10.1017/S0033291719000357

**Published:** 2020-02

**Authors:** Andries T. Marees, Dirk J. A. Smit, Jue-Sheng Ong, Stuart MacGregor, Jiyuan An, Damiaan Denys, Florence Vorspan, Wim van den Brink, Eske M. Derks

**Affiliations:** 1 Department of Psychiatry, Amsterdam UMC, Amsterdam Neuroscience, University of Amsterdam, Meibergdreef 9, Amsterdam, The Netherlands; 2 Translational Neurogenomics Group, QIMR Berghofer Medical Research Institute, Brisbane, Australia; 3 Statistical Genetics, QIMR Berghofer Medical Research Institute, Brisbane, Australia; 4 Assistance Publique – Hôpitaux de Paris, Hôpital Fernand Widal, Département de Psychiatrie et de Médecine Addictologique, 200 rue du Faubourg Saint Denis, 75010 Paris, France; 5 Inserm umr-s 1144, Université Paris Descartes, Université Paris Diderot, 4 avenue de l'Observatoire, 75006 Paris, France

**Keywords:** Alcohol consumption, genetic correlation, genetics, socioeconomic status, substance use

## Abstract

**Background.** Frequency and quantity of alcohol consumption are metrics commonly used to measure alcohol consumption behaviors. Epidemiological studies indicate that these alcohol consumption measures are differentially associated with (mental) health outcomes and socioeconomic status (SES). The current study aims to elucidate to what extent genetic risk factors are shared between frequency and quantity of alcohol consumption, and how these alcohol consumption measures are genetically associated with four broad phenotypic categories: (i) SES; (ii) substance use disorders; (iii) other psychiatric disorders; and (iv) psychological/personality traits.

**Methods.** Genome-Wide Association analyses were conducted to test genetic associations with alcohol consumption frequency (*N* = 438 308) and alcohol consumption quantity (*N* = 307 098 regular alcohol drinkers) within UK Biobank. For the other phenotypes, we used genome-wide association studies summary statistics. Genetic correlations (*r*_g_) between the alcohol measures and other phenotypes were estimated using LD score regression.

**Results.** We found a substantial genetic correlation between the frequency and quantity of alcohol consumption (*r*_g_ = 0.52). Nevertheless, both measures consistently showed opposite genetic correlations with SES traits, and many substance use, psychiatric, and psychological/personality traits. High alcohol consumption frequency was genetically associated with high SES and low risk of substance use disorders and other psychiatric disorders, whereas the opposite applies for high alcohol consumption quantity.

**Conclusions.** Although the frequency and quantity of alcohol consumption show substantial genetic overlap, they consistently show opposite patterns of genetic associations with SES-related phenotypes. Future studies should carefully consider the potential influence of SES on the shared genetic etiology between alcohol and adverse (mental) health outcomes.

## Introduction

While alcohol is widely consumed worldwide, socially accepted, and legally available in many cultures, its effects on individuals and society are heavily debated. On the one hand, low-volume alcohol consumption does not seem to affect mortality significantly compared to abstinence or occasional drinking (Stockwell *et al*., 2016). On the other hand, alcohol consumption is robustly associated with the risk of over 200 diseases (Rehm *et al*., 2010; Roerecke and Rehm, 2012; World Health Organisation, 2014). Furthermore, 5.1% of the global burden of disease and injury, measured in disability-adjusted life years (DALYs), is attributable to alcohol consumption (World Health Organisation, 2014) with accidents and injuries causing 30% of the DALYs and various chronic diseases causing the remaining 70% of the DALYs (World Health Organisation, 2014). Most alcohol-related premature deaths are caused by the consumption of harmful levels of alcohol and alcohol dependence (AD) (Rehm *et al*., 2013).

Considering the potentially harmful effects of alcohol consumption, especially when consumed in large quantities (e.g. Stahre *et al*., 2014), it is important to monitor alcohol consumption behaviors. To assess problematic consumption of alcohol in the general population, current alcohol consumption is a useful metric as it allows for a comparison to the guidelines for low-risk alcohol consumption (e.g. ⩽14 alcoholic beverages per week, in most countries the threshold is lower for women than for men; CMOs, 2016, Kalinowski and Humphreys, 2016). It has also been proposed to replace alcohol use disorder diagnostics by simple alcohol consumption metrics with treatments of problematic or excessive alcohol consumption directed at the reduction of alcohol use (Nutt and Rehm, 2014). The benefit of assessing levels of alcohol consumption by questionnaires is that it is cost-effective which allows the inclusion of larger study samples. Furthermore, fast and low-cost assessment allows for the inclusion of more people in treatment and may therefore narrow the current treatment gap for people with an alcohol use disorder (Kohn *et al*., 2004; Tuithof *et al*., 2016). The diagnostic value of alcohol consumption metrics is exemplified by the presence of considerable phenotypic correlations with, for example, AD (Kendler *et al*., 2010). Two widely used measures to monitor alcohol consumption in the general population are ‘alcohol consumption frequency’ (i.e. the number of days on which alcohol is consumed per week) and ‘alcohol consumption quantity’ (i.e. the number of standard alcoholic drinks consumed per week).

Alcohol consumption frequency and alcohol consumption quantity have a high phenotypic correlation (based on UK Biobank data; Sudlow *et al*., 2015), and have been used interchangeably in some epidemiological studies (Berggren and Sutton, 1999, Heckley *et al*., 2017). However, various studies suggest that alcohol consumption quantity and alcohol consumption frequency have different and sometimes opposing effects on health. Differences in effect have been shown for cardiovascular diseases, cancers, and various mental health conditions. For example, high alcohol consumption quantity has been related to an elevated risk of all-cause mortality, cardiovascular disease, depression, and insomnia, to reduced cerebellar blood flow, and a higher risk of cancer in men. In contrast, high alcohol consumption frequency (restricted to low-to-moderate consumption quantity), has previously been related to a reduced risk of cardiovascular disease, type 2 diabetes, improved cerebellar blood flow, and an increased risk of cancer in women (Conigrave *et al*., 2001; Stein and Friedmann, 2005; Breslow and Graubard, 2008; Christie *et al*., 2008; Breslow *et al*., 2011; Ronksley *et al*., 2011; Piano, 2017).

The opposing effects on the health of these alcohol consumption patterns may be explained by their differential association with specific life circumstances. For example, research suggests that people with a high socio-economic status (SES; e.g. assessed based on income and education) tend to drink more frequently and consume a larger total amount than people with a lower SES (Heckley *et al*., 2017), but consume on average less per drinking occasion. Furthermore, alcohol consumption is known to be related to a broad range of phenotypes, including substance use disorders (Grucza and Bierut, 2006; Kendler *et al*., 2010), other psychiatric disorders (Clark *et al*., 1997; Kessler *et al*., 1997; Khoury *et al*., 2010), and personality/psychological traits (Hicks *et al*., 2012; Turiano *et al*., 2012; Hakulinen *et al*., 2015). The complex relation of the alcohol consumption with SES (Staff *et al*., 2008; Sudlow *et al*., 2015; Collins, 2016; Heckley *et al*., 2017) is especially interesting, because SES itself also has a known relation with substance use disorders, psychiatric disorders, and personality/psychological traits (e.g. Hiscock *et al*., 2012; von Stumm and Plomin, 2015; Russell *et al*., 2016). However, epidemiological studies that investigate associations of alcohol consumption with various other phenotypes do not provide insight into the underlying biological mechanisms which explain differential patterns of phenotypic associations for frequency and quantity of alcohol consumption.

Twin studies show that 50–60% of the phenotypic variation in alcohol consumption quantity (Swan *et al*., 1990), and alcohol use disorders (Mbarek *et al*., 2015; Verhulst *et al*., 2015) are heritable. Furthermore, several studies have explored which genetic variants contribute to alcohol consumption quantity using Genome-Wide Association analysis (GWAS). The most recent and largest study of alcohol consumption to date has identified 14 significant loci (Clarke *et al*., 2017). The aldehyde dehydrogenases (*ALDH*) and alcohol dehydrogenases (*ADH*) gene clusters have proven to be robust findings as they were consistently found by various alcohol consumption quantity GWAS (Schumann *et al*., 2016; Clarke *et al*., 2017; Jorgenson *et al*., 2017). Moreover, the *ADH* cluster has also been found to play a role in AD (Gelernter *et al*., 2014*a*; Walters *et al*., 2018). Individually, single nucleotide polymorphisms (SNPs), identified by GWAS, explain a very small proportion of the variation in alcohol consumption quantity, as is the case for most complex traits (Manolio *et al*., 2009; Clarke *et al*., 2017). However, the SNP-based heritability of alcohol consumption quantity is estimated at 13–18% (Vrieze *et al*., 2013; Clarke *et al*., 2017), indicating that collectively GWAS SNPs explain a modest proportion of the variation in alcohol consumption quantity. Until now, no GWAS studies have been published on alcohol consumption frequency.

The reason that genetic variants that are genome-wide significantly associated with alcohol consumption only explain a modest proportion of the heritability is because alcohol consumption, like most other complex traits, has a polygenic genetic architecture. Therefore, the total heritability is distributed over thousands of variants of small effect (Visscher *et al*., 2017). A method that estimates genetic overlap by utilizing the information of all genetic variants in a GWAS, including the ones below the stringent significance threshold, is genetic correlation. Compared to epidemiological studies, which allow investigation of phenotypic correlations, genetic correlation analysis has the benefit of providing insight into the extent to which genetic risk factors are shared between traits. Previous studies which used this technique demonstrated genetic overlap between various psychiatric traits which were traditionally not seen as closely related (Anttila *et al*., 2018), and demonstrated genetic overlap between various substance use traits (Nivard *et al*., 2016). Furthermore, a study by Hill *et al*. (2016) demonstrated that heritability of SES is captured by GWAS, and showed substantial genetic correlations with various complex traits (Hill *et al*., 2016). Genetic correlation analysis, therefore, also allows for the investigation of the complex relation between the quantity and frequency of alcohol consumption with SES and other complex traits, on a genetic level.

The current study aims to elucidate to what extent genetic factors are shared between quantity (in regular drinkers) and frequency of alcohol consumption. In addition, we investigate genetic correlations of alcohol consumption quantity and frequency with traits in four phenotypic categories: SES, substance use disorders, other psychiatric disorders and personality/psychological traits. These genetic correlations will provide a better insight into the etiology of different alcohol consumption behaviors and their relation to other phenotypic traits.

## Methods

### UK Biobank data

Data used for the alcohol consumption frequency and alcohol consumption quantity GWAS were obtained from UK Biobank (http://www.ukbiobank.ac.uk) (Sudlow *et al*., 2015). The UK Biobank resource was established by the Wellcome Trust medical charity, Medical Research Council, Department of Health, Scottish Government and the Northwest Regional Development Agency. It is a major resource with the aim of improving the prevention, diagnosis and treatment of a wide range of health problems. UK Biobank recruited over 500 000 individuals aged 40–69 years between 2006 and 2010 from across the UK, and collected information on >2000 phenotypes (Sudlow *et al*., 2015).

Information on alcohol consumption frequency was obtained through a self-report questionnaire (UK Biobank field ID: 1558; description: Alcohol consumption frequency), which contained seven ‘frequency’ categories, from ‘never’ to ‘daily or almost daily’, individuals also had the option to fill out: ‘prefer not to tell’. Alcohol consumption frequency was assessed in a total of 501 731 subjects. In those who indicated to drink at least once or twice a week (i.e. regular drinkers), also information on alcohol consumption quantity was assessed (*N* = 348 039). Occasional drinkers, defined as drinking less than once per week (Stockwell *et al*., 2016), were excluded from our analyses, since the nature of our data prevented an accurate assessment of their weekly alcohol consumption quantity. In addition, occasional drinkers are known to underestimate their personal alcohol consumption (Stockwell *et al*., 2014; Stockwell *et al*., 2016). The assessment of alcohol consumption quantity was made using the average weekly alcohol intake for five general alcohol beverage classes: 1568 (red wine) 1578 (champagne plus white wine), 1598 (spirits), 1558 (beer plus cider), and 1608 (fortified wine). The following item was used ‘In an average WEEK, how many glasses of -class of alcohol- would you drink?’ The total units of alcohol were calculated by multiplying the number of glasses with a factor depending on the class of alcohol, a procedure similar to the one used by Clarke *et al*. (2017). The factors we used for the multiplication were 1.67 (red wine and champagne/white wine), 2.3 (beer), 1 (spirit) and 2.25 (fortified wine). Individuals with an alcohol consumption quantity deviating >5 s.d. from their sex-specific mean were excluded from our analyses.

### GWAS

SNPs with imputation quality <0.6 and MAF <0.001 were excluded from the association analysis. After genetic QC and exclusion of ethnic outliers, we included 438 308 individuals who were genetically identified to be of white British ancestry in the alcohol consumption frequency GWAS and 307 098 individuals in the alcohol consumption quantity GWAS (i.e. a subset of the alcohol consumption frequency sample). The detailed information regarding the UK biobank genotyping and QC for our analyses was described in Ong *et al*. (2018).

Due to the large number of related individuals in the UK Biobank cohort, the GWAS for alcohol consumption frequency and alcohol consumption quantity were performed using BOLT-LMM, which is a linear mixed model framework that explicitly models the genetic relatedness within the sample (Loh *et al*., 2015). More specifically, GWAS analyses were performed using the BOLT-LMM v2.3 package, with age, sex, and the top ten ancestral principal components fitted as covariates in the model.

### Summary statistics

Data from the other phenotypes used in the current study consisted of summary statistics from previously conducted GWAS meta-analyses, all of which were based on large samples, including UK biobank data (http://www.nealelab.is/blog/2017/7/19/rapid-gwas-of-thousands-of-phenotypes-for-337000-samples-in-the-uk-biobank). We included 41 other phenotypic traits: eight substance use disorder traits, 11 other psychiatric traits, 13 personality and psychological traits, and nine SES-related measures (Table 1). Detailed information about the samples included in this study is provided in Supplementary Table 1.

**Table 1.** Overview of the GWAS samples used in the current study

Category	Trait	*N*	PubMed or bioRxiv id	Citation	Source*
Alcohol consumption	Frequency	438 870	NA	NA	Own work
	Quantity	307 098	NA	NA	
Substance use traits	Alcohol dependence	10 206 cases, 28 480 controls	bioRxiv 257311	Walters *et al.*, (2018)	Obtained from authors
	Cannabis lifetime	32 330	PubMed 27023175	Stringer *et al.*, (2016)	
	Cannabis dependence	8754	PubMed 27028160	Sherva *et al.*, (2016)	
	Cocaine dependence	4769	PubMed 23958962	Gelernter *et al.*, (2014*b*)	
	Cigarettes per day, current	38 181	PubMed 20418890	Furberg *et al.*, (2010)	PGC
	Cigarettes per day, previously	78 291	NA	NA	UKBB GWAS Manifest
	Current tobacco smoking	337 030	NA	NA	
	Time from wake till the first cigarette	23 265	NA	NA	
Psychiatric traits	Major depressive disorder	59 851 cases and 113 154 controls	bioRxiv 167577	Wray *et al.*, (2018)	Obtained from authors
	Depressive symptoms	161 460	PubMed 27089181	Okbay *et al.*, (2016*a*)	SSGAC
	Schizophrenia	36 989 cases, 113 075 controls	PubMed 25056061	Ripke *et al.*, (2014)	PGC
	Bipolar disorder	11 974 cases, 51 792 controls	PubMed 21926972	Sklar *et al.*, (2011)	
	Autism	18 381 cases, 27 969	bioRxiv 224774	Grove *et al.*, (2019)	
	OCD	2688 cases, 7037 controls	PubMed 28761083	Arnold *et al.*, (2018)	
	PTSD	9500 (25% cases)	PubMed 28439101	Duncan *et al.*, (2018)	
	Anxiety	>18,000	PubMed 26754954	Martin *et al.*, (2017)	
	ADHD adult	20 183 cases, 35 191 controls	bioRxiv 145581	Demontis *et al.*, (2019)	
	ADHD child	17 666	PubMed 27663945	Middeldorp *et al.*, (2016)	
	Child aggression	18 988	PubMed 26087016	Pappa *et al.*, (2016)	
Personality/ psychological traits	Loneliness	10 760	PubMed 27629369	Gao *et al.*, (2017)	PGC
	Loneliness/isolation	58 752 cases, 273 511 controls	NA	NA	UKBB GWAS Manifest
	Risk-taking	82 808 cases, 243 013 controls	NA	NA	
	Overall Health rating	336 020	NA	NA	
	Sensitivity/ hurt feelings	182 340 cases, 145 492 controls	NA	NA	
	Tiredness	108 976	PubMed 28194004	Deary *et al.*, (2018)	SSGAC
	Neuroticism	329 000	PubMed 29255261	Luciano *et al.*, (2018)	
	Subjective well-being	298 420	PubMed 27089181	Okbay *et al.*, (2016*a*)	
	Insomnia	113 006	PubMed 28604731	Hammerschlag *et al.*, (2017)	CTG
	Verbal and numerical reasoning	168 033	PubMed 29844566	Davies *et al.*, (2018)	CCACE
	General cognitive functioning	282 014	PubMed 29844566	Davies *et al.*, (2018)	
	Openness to new experience	17 375	PubMed 27918536	Lo *et al.*, (2017)	GCP
	Conscientiousness	17 375	PubMed 27918536	Lo *et al.*, (2017)	
Social economic status	Mothers age of death	199 690	NA	NA	UKBB GWAS Manifest
	Fathers age of death	248 726	NA	NA	
	Age of first birth	123 846	NA	NA	
	College or university degree	334 070	NA	NA	
	Age of completed full-time education	226 899	NA	NA	
	Current employment	336 252	NA	NA	
	Educational attainment	293 723	PubMed 27225129	Okbay *et al.*, (2016*b*)	SSGAC
	Social deprivation	112 005	PubMed 27818178	Hill *et al.*, (2016)	CCACE
	Household income	96 900	PubMed 27818178	Hill *et al.*, (2016)	

CCACE, Centre for Cognitive Ageing and Cognitive Epidemiology (http://www.ccace.ed.ac.uk/node/335); CTG, Complex Trait Genetics (https://ctg.cncr.nl/software/summary_statistics); GCP, Genetics of Personality Consortium (http://www.tweelingenregister.org/GPC/); PGC, Psychiatric Genetics Consortium (https://www.med.unc.edu/pgc/results-and-downloads); SSGAC, Social Science Genetic Association Consortium (https://www.thessgac.org/data); UKBB GWAS Manifest (https://docs.google.com/spreadsheets/d/1b3oGI2lUt57BcuHttWaZotQcI0-mBRPyZihz87Ms_No/edit#gid=1209628142)

## Cross-trait linkage disequilibrium (LD) score regression

LD score regression is a method based on the assumption that an estimated SNP effect-size includes effects of all SNPs in LD (a measure of non-random association between alleles at different loci at the same chromosome in a given population) with that SNP. SNPs that tag (represent due to LD) many other SNPs will have a higher probability of tagging causal genetic variants compared to SNPs that tag few other SNPs. The LD-score is a measure of the amount of genetic variation tagged by a particular SNP within a specific population. Therefore, SNPs with a higher LD-score have, on average, stronger effect sizes than SNPs with lower LD-scores (Bulik-Sullivan *et al*., 2015*b*). Thus, if the effect size from the association analysis is regressed against the LD-score for each SNP, the slope of the regression line provides an estimate of the proportion of variance explained by all analyzed SNPs (Bulik-Sullivan *et al*., 2015*b*). An extension of this method, which allows for the estimation of genetic correlation, is cross-trait LD score regression (Bulik-Sullivan *et al*., 2015*a*). The genetic correlation is estimated using the slope from the regression of the product of *z*-scores from the GWAS on the LD-score. This estimate represents the genetic covariation between the two traits based on all polygenic effects captured by SNPs. Note that genetic correlation is a stronger condition than pleiotropy: a pleiotropic relation only entails that two traits are influenced by the same genetic variant, but for a genetic correlation the directions of effect must be consistently aligned, across the genome, either in the same direction (a positive genetic correlation) or in the opposite direction (a negative genetic correlation) (Bulik-Sullivan *et al*., 2015*a*). The cross-trait LD score regression analyses in the current study were performed using 1 215 002 SNPs that were present in the HapMap 3 reference panel with a 1000 genomes project EUR (European) MAF >5%, with the exclusion of the MHC region.

Significant genetic correlations were identified by applying a FDR 0.05 threshold over all tests. For visualization, we used the R-library ‘ggplot2’.

## Results

Alcohol consumption frequency in all drinkers and alcohol consumption quantity in regular drinkers displayed strong polygenic signals (Supplementary Table 1). The genetic correlation between these alcohol consumption metrics was 0.52 (*p* = 1.31·10^−131^), while the phenotypic correlation was 0.62 (*p* < 0.001).

**Fig. 1.**
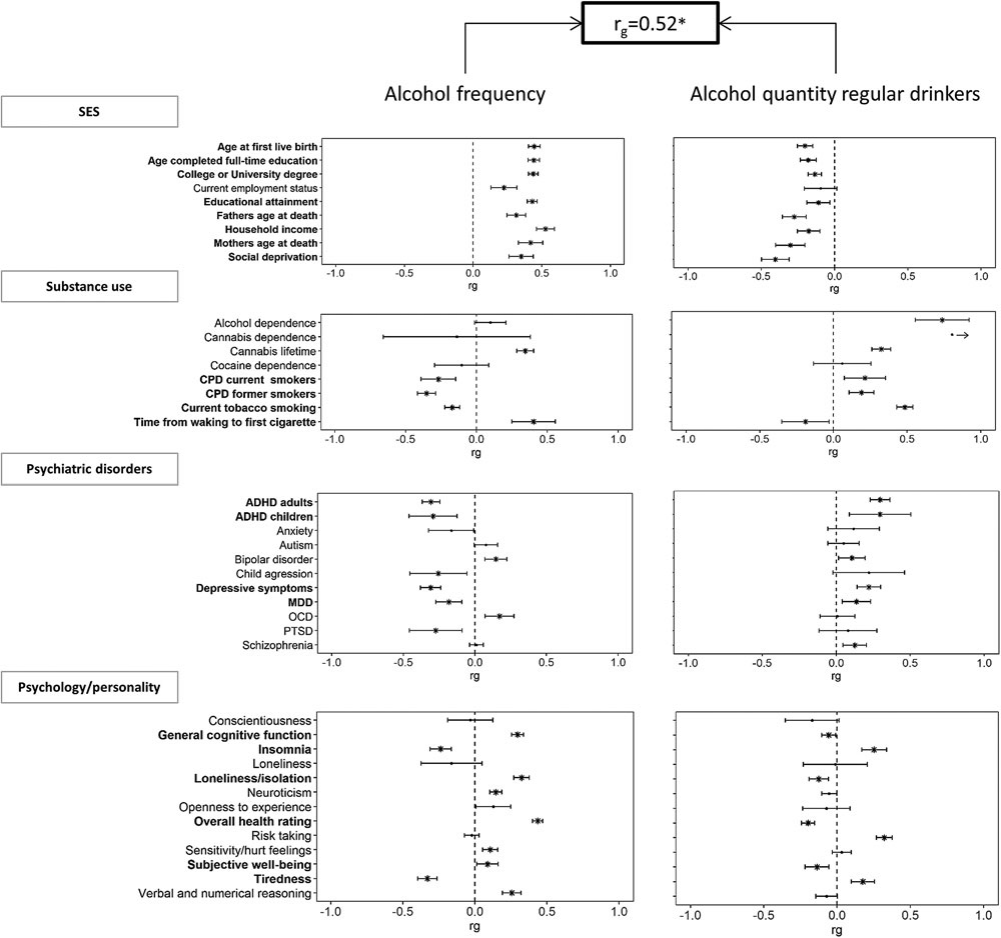
Genetic overlap between alcohol frequency (left panels) and alcohol quantity (right panels) against four categories of traits; SES, substance use disorders, psychiatric disorders, and personality/psychological traits. Traits printed in bold show opposite directions of effect. The error bars represent 95% confidence intervals, an arrow towards one indicates confidence interval >1 or −1, asterisks indicate significant associations (FDR-adjusted *p*-value <0.05). Social deprivation scores were reversed so that higher social deprivation/Townsend indicates higher SES.

Despite the positive genetic correlation between the two alcohol consumption measures, external phenotypes often showed significant genetic correlations with both alcohol consumption measures in opposite directions. Traits that show significant correlations with both traits, but in opposite directions, are indicated in bold in Fig. 1. Since the GWAS of alcohol consumption quantity was performed in a subset of the sample of the GWAS of alcohol consumption frequency, we investigated the potential influence of this selection. We conducted a sensitivity analysis by performing a GWAS of alcohol consumption frequency in regular drinkers only (the same subset that was used for the alcohol consumption quantity analysis). The opposite pattern of genetic correlations remained largely similar, although for loneliness/isolation we observed a sign flip of the genetic correlation (Supplementary Fig. 2).

In the full analysis, frequency of alcohol consumption showed significant genetic correlations (FDR-adjusted *p*-value <0.05) with 31 of the 40 other phenotypic traits: nine SES traits, five substance use disorder traits, eight other psychiatric disorder traits, and nine psychological/personality-related traits. Genetic correlations with measures of SES were consistently positive, i.e. frequent drinking was genetically associated with higher levels of SES. Frequent drinking was genetically negatively associated with tobacco smoking, as indicated by four different smoking measures, i.e. frequent drinking was associated with lower levels of smoking. In contrast, a positive genetic correlation was found between frequency of alcohol consumption and the risk of lifetime cannabis use. We did not observe evidence for a significant genetic correlation between alcohol consumption frequency and AD. The pattern of genetic correlations with other psychiatric disorders was somewhat inconsistent. We observed that frequency of consumption was genetically associated with a reduced risk of ADHD, Major Depressive disorder (MDD), and depressive symptoms, while the genetic correlation with bipolar disorder was positive. A higher genetic predisposition for frequency of alcohol consumption was associated with reduced ratings of insomnia and self-reported tiredness, increased levels of verbal and numerical reasoning, general cognitive functioning, subjective well-being, overall health, loneliness, neuroticism, and an increased vulnerability to getting feelings hurt.

Among regular drinkers, the quantity of alcohol consumption showed significant genetic correlations (FDR-adjusted *p*-value <0.05) with 27 of the 40 phenotypic traits: eight SES traits, six substance use disorder traits, six psychiatric disorder traits, and seven psychological/ personality-related traits (see Fig. 1). The genetic correlations of alcohol consumption quantity and different measures of SES were consistently negative; high quantity of alcohol consumption was genetically associated with low SES. Alcohol consumption quantity was genetically positively associated with the number of cigarettes smoked per day, AD, and lifetime cannabis use. We observed that a higher genetic risk for increased alcohol consumption quantity was associated with an increased genetic risk for schizophrenia, bipolar disorder, depression, and ADHD. Furthermore, we observed a positive genetic correlation with insomnia, risk-taking, and tiredness ratings, whereas negative genetic correlations were found with subjective well-being, general cognitive function, loneliness isolation, and self-reported health.

Thus, alcohol consumption frequency in all subjects and alcohol consumption quantity in regular drinkers often showed the opposite direction of effect (sign of correlation coefficient) on all four categories of phenotypes (see Fig. 1 and Supplementary Fig. 1), as highlighted by the pattern of association with SES.

## Discussion

The overarching aim of this study was to explore the genetic correlation between alcohol consumption frequency and alcohol consumption quantity in regular drinkers, and to examine the genetic correlations of these alcohol consumption measures against 41 other phenotypic traits. We included traits from four broad categories: SES, substance use disorders, other psychiatric disorders, and personality/personality traits, using GWAS summary statistics of studies comprising 4000~ 100 000 subjects. We identified a phenotypic and genetic correlation between alcohol consumption frequency and alcohol consumption quantity of 0.62 (*p* < 0.001) and 0.52 (*p* = 1.31·10^−131^). Although these are substantial correlations, it clearly demonstrates that these two metrics cannot be used interchangeably, confirming previous epidemiological evidence (Berggren and Sutton, 1999; Heckley *et al*., 2017). Furthermore, alcohol consumption frequency and alcohol consumption quantity showed significant genetic correlations with many of the other examined phenotypic traits, but these genetic correlations often showed effects in opposing directions.

In the categories ‘substance use disorders’, ‘other psychiatric disorders’, and ‘personal/psychological traits’, 14 phenotypic traits showed opposite directions of effect to the alcohol measures central in this study. Alcohol consumption quantity (in regular drinkers) was genetically associated with psychopathology, whereas this was generally not the case for alcohol consumption frequency, suggesting that these alcohol consumption metrics indeed measure different aspects of drinking behavior with different genetic risk profiles. These opposite effects were most consistently found in the genetic correlations with SES-related measures. Epidemiological studies suggest that high SES is related to a higher frequency of alcohol consumption, whereas low SES is related to higher alcohol consumption quantity (Casswell *et al*., 2003; Huckle *et al*., 2010; Giskes *et al*., 2011). Our results show these opposing phenotypic associations are at least partly explained by opposite associations at the genetic level.

The observation that SES has a positive genetic correlation with alcohol consumption frequency and negative genetic correlation with alcohol consumption quantity may suggest that SES plays a mediating role in the pattern of genetic correlation we observe between these alcohol consumption measures and the other phenotypes. This hypothesis is supported by the observation that individuals with lower SES seem to bear a disproportionate burden of negative alcohol-related consequences (Collins, 2016). Previously, genetic correlations have been reported between SES and various behavioral phenotypes (Hill *et al*., 2016), including smoking, various psychiatric disorders, and psychology/personality-related traits. Exploration of the patterns observed in the current study with those found by Hill *et al*., reveals noteworthy similarities. Six of the seven phenotypes that showed significant correlation with SES in the study by Hill *et al*. (i.e. educational attainment, intelligence, schizophrenia, smoking, MDD, and bipolar disorder) were correlated with alcohol frequency and alcohol quantity in opposite directions in the current study, where high SES and high frequency of alcohol consumption had consistent signs. The only exception was formed by neuroticism, which in contrast to the other results in the comparison, had an opposing direction between high SES and high alcohol consumption frequency. This observation further supports our findings that variability in alcohol consumption frequency and quantity is partly explained through the influence of SES but in opposite directions.

If SES mediates many of the genetic correlations between alcohol consumption traits and other traits, we expect that these traits show opposite effects in their correlation between alcohol consumption frequency and quantity as well. Indeed, the various smoking parameters showed opposite patterns genetic correlations between frequency and quantity of alcohol consumption. Increased alcohol consumption quantity was genetically associated with increased levels of smoking, whereas the increased frequency of alcohol consumption was genetically associated with reduced levels of smoking. The latter seems somewhat counterintuitive, as previous studies have shown a positive phenotypic correlation between smoking and frequency of drinking (Jiang and Ling, 2013; Lo *et al*., 2013). Furthermore, those with low SES drink less frequently than those with high SES. Since low SES increases the susceptibility to smoking (Hiscock *et al*., 2012), it seems plausible that the negative genetic association between frequency of alcohol consumption and smoking is mediated by SES. Cannabis lifetime use showed a positive genetic correlation with both alcohol consumption metrics, which seems to be supported by the phenotypic/epidemiological literature (Subbaraman and Kerr, 2015). Previous research indicates that adolescents from families of higher SES are more likely to experiment with cannabis, which is also suggested by a recent genetic correlation study (Legleye *et al*., 2012; Pasman *et al*., 2018), possibly mediating this relation. The positive genetic correlation of alcohol consumption quantity with cannabis lifetime, conflicts with our SES hypothesis, and might be explained through other factors such as risk-taking behavior (de Haan *et al*., 2015; Pasman *et al*., 2018).

With regard to other psychiatric disorders, we found that alcohol consumption frequency and alcohol consumption quantity were correlated with ADHD, depressive symptoms and MDD in opposite directions. Interestingly, the negative genetic association between alcohol consumption frequency and risk of both ADHD and depression is not supported by recent (endo)phenotypic studies, which found no evidence of association of alcohol consumption frequency with ADHD and a positive association with MDD (Weafer *et al*., 2011; Edwards *et al*., 2014). However, previous research has indicated a phenotypic association of low SES with the presence of ADHD and MDD (Gavin *et al*., 2010; Russell *et al*., 2016). This may imply that genetic variants associated with low SES make individuals more susceptible to develop ADHD and MDD.

The benefit of investigating genetic correlations from GWAS over phenotypic correlations obtained by conventional epidemiological studies are numerous. Genetic correlations provide information on the genetic similarity of phenotypic traits. Therefore, this method allows investigation of whether traits which are in high phenotypic correlation with each other are also genetically similar, e.g., frequency and quantity of alcohol consumption. A high genetic correlation might suggest shared causal genes and shared biological pathways between the traits, while a low genetic correlation would suggest that a high phenotypic correlation is caused by independent biological or environmentally determined mechanisms. This information can provide additional insights into biology underlying co-morbidity and disease risk compared to epidemiological studies. Furthermore, due to the ability of genetic correlation to investigate the genetic similarity between traits, this method can aid in finding alternative phenotypes, which are genetically similar to an outcome of interest, but are less expensive to assess and thus more suited for large scale studies. For example, based on our results it could be concluded that alcohol consumption quantity but not alcohol consumption frequency would be a valid proxy for AD, since AD showed a positive genetic correlation with quantity but not with frequency of alcohol consumption. This finding is in line with results from bivariate twin modeling which has shown a shared heritability of 0.63 (Whitfield *et al*., 2004) between AD and quantity of alcohol consumption.

Taken together, the current study suggests a possible mediation of SES in the (genetic) correlation between alcohol consumption measures and other phenotypic traits. However, genetic correlations between SES and substance use disorders, other psychiatric disorders, and personality/psychology-related phenotypes are complex in itself and can be explained in at least three ways. The first explanation is that two phenotypes are genetically correlated due to the influence of genetic variants with pleiotropic effects of the same net direction (e.g. alcohol consumption frequency ß shared genetic effects (G) à educational attainment). The second form of genetic correlation emerges if a certain phenotype is influenced by genetic variants, and that phenotype has a direct causal relationship with another phenotype (e.g. G à educational attainment à alcohol consumption frequency) (Solovieff *et al*., 2013). The third form of genetic correlation can occur through gene–environment interaction (e.g. G à specific environment à alcohol consumption frequency and educational attainment). Since our study was designed to only evaluate the correlation structure between these variables, we are unable to definitively untangle different modes of pleiotropy.

We have shown significant genetic correlations between SES and measures of alcohol consumption. SES is a complex construct that is influenced by other traits such as intelligence and personality traits (Hill *et al*., 2016). This raises the possibility that the alcohol consumption measures central in the current study are being mediated by phenotypic traits which influence the SES of the individual. Therefore, genetic risk variants which directly influence phenotypic traits which affect SES, might be picked up by a GWAS investigating alcohol consumption. The correct interpretation of such findings is challenging for researchers, as significant loci are normally interpreted within the perspective of plausible biological pathways of the trait under investigation. The current study, and other genetic correlation studies, can help to widen the perspective of researchers outside the scope of biological mechanisms causally affecting a single phenotype of interest. Deciphering the meaning of future GWAS findings in light of the vast interdependency of phenotypes can aid our understanding of the complex genetic architecture of trait and disease and their underlying causal mechanisms.

Our results may have been influenced by selection bias. The response rate in the UK Biobank sample is <5%, and this subset may be an inaccurate representation of the UK population. Previous studies have indicated that the UK Biobank sample is healthier than the general population, suggesting that selection or selection bias may have had some impact on our findings regarding SES, education, and mental health traits (Knudsen *et al*., 2010; Fry *et al*., 2017; Davis *et al*., 2018). In addition, the selection of regular drinkers could potentially make our study sensitive to collider bias. Collider bias occurs when two variables independently influence a third variable, and that third variable is conditioned upon. Collider bias can lead to biased estimates of associations (Munafo *et al*., 2018). Before and after restricting both alcohol consumption measures the general pattern of the opposite genetic correlations remained. The exception to this was loneliness/isolation, where we observed a sign flip of the genetic correlation after restricting alcohol consumption frequency to regular drinkers. However, selecting regular drinkers for the GWAS of alcohol consumption is not likely to have caused collider bias, since regular drinking is not ‘caused’ by frequency of alcohol consumption, namely, regular drinking is a dichotomized version of frequency of alcohol consumption. Likewise, due to its arithmetic relation with frequency of alcohol consumption (see methods), the quantity of alcohol consumption is not fully explained by a causal influence of alcohol frequency. Nevertheless, we cannot fully exclude the possibility of a collider being present, nor would we want to exclude this possibility. Previous alcohol consumption GWAS have used similar selection strategies (e.g. Schumann *et al*., 2016) and may therefore also be sensitive to selection or collider bias. It should therefore be emphasized that our results are only representative for the population of regular drinkers. Due to the complex pattern of associations between alcohol consumption quantity/frequency with external variables (e.g. SES) observed in our study, it is clear that statements such as ‘one glass of red wine is good for your health’ are a simplification of the true picture.

The findings and conclusions of this study should be interpreted in view of some key limitations. An important limitation is that our estimates of genetic correlations are based on LD score regression using only common SNPs. In family studies, the information on all genetic variants is captured, not just common SNPs. Therefore, family studies estimate the total genetic correlation. If effects are differently correlated among common variants than among rare variants the total genetic correlation can deviate from the common SNP-based genetic correlation (Bulik-Sullivan *et al*., 2015*a*). Furthermore, while alcohol consumption frequency was assessed in a general population sample (regular drinkers, occasional drinkers and teetotalers), alcohol consumption quantity was assessed only in participants who drink regularly. This led to a significantly smaller sample for alcohol consumption quantity, and a reduction in power to find significant genetic correlations relative to frequency of alcohol consumption. Moreover, this study assessed current alcohol intake, which might have led to misclassification of some subjects in regards to the genetic risk factors they carry for alcohol consumption, since alcohol intake is not stable over time (Kerr *et al*., 2002). Furthermore, despite the comparatively large sample sizes of the alcohol and non-alcohol consumption GWAS in the current study, some of our analyses may still be underpowered to reliably detect genetic correlations. In addition, this study showed merely genetic correlations, it is important to realize that correlations do not equal causation. To fully evaluate causality, Mendelian Randomization (MR) studies ought to be conducted (Lawlor *et al*., 2008), although at present the genetic instruments for most phenotypes investigated in these analyses are not sufficient to allow well-powered MR analyses. The individual genetic correlations should therefore be interpreted carefully with these limitations in mind. Furthermore, the possible mediating role of SES on the alcohol consumption measures might obscure their true genetic correlation to non-SES traits, which has not been tested in the present study.

## Conclusion

We have shown that although frequency and quantity of alcohol consumption showed substantial genetic overlap, they consistently show opposite patterns of genetic correlations with SES-related phenotypes. Our findings provide novel insights into the genetic architecture shared between alcohol consumption and phenotypes of four broad categories (SES, substance use disorders, other psychiatric disorders and psychology/personality traits) and hints SES as a potential mediator of the relationship. The latter indicates that future studies should carefully consider the potential influence of SES on the shared genetic etiology between alcohol and adverse conditions.

## Supplementary material

For supplementary material accompanying this paper visit http://dx.doi.org/10.1017/S0033291719000357. click here to view supplementary material
