# Supplementary material for: Potential influence of socioeconomic status on genetic correlations between alcohol consumption measures and mental health
Source: Psychol Med. 2019 Mar 15;50(3):484–98. doi: 10.1017/S0033291719000357 (PMC7083578; doi:10.1017/S0033291719000357)
Supplement: Supplementary file 1 [file S0033291719000357sup001.docx]

**Supplementary information for:**

**Potential influence of socio-economic status on genetic correlations between alcohol consumption measures and mental health**

Andries T. Marees, Dirk J.A. Smit, Jue-Sheng Ong, Stuart MacGregor, Jiyuan An, Damiaan Denys, Florence Vorspan, Wim van den Brink, Eske M. Derks

Supplementary Table 1. Overview of the LD score metrics for alcohol consumption data

| **Trait** | **Mean χ2 statistic** | **Intercept (SE)** | **Ratio (SE)** | **Heritability Z-score** | **Heritability** |
| --- | --- | --- | --- | --- | --- |
| Alcohol consumption frequency | 1.76 | 1.07 (0.0116) | 0.09 (0.0153) | 24.2 | 0.08 (0.0033) |
| Alcohol consumption frequency (in regular drinkers): | 1.36 | 1.03 (0.0078) | 0.09 (0.0219) | 18.7 | 0.043 (0.0023) |
| Alcohol consumption quantity (in regular drinkers): | 1.37 | 1.03 (0.0097) | 0.06 (0.0217) | 19.4 | 0.068 (0.0035) |


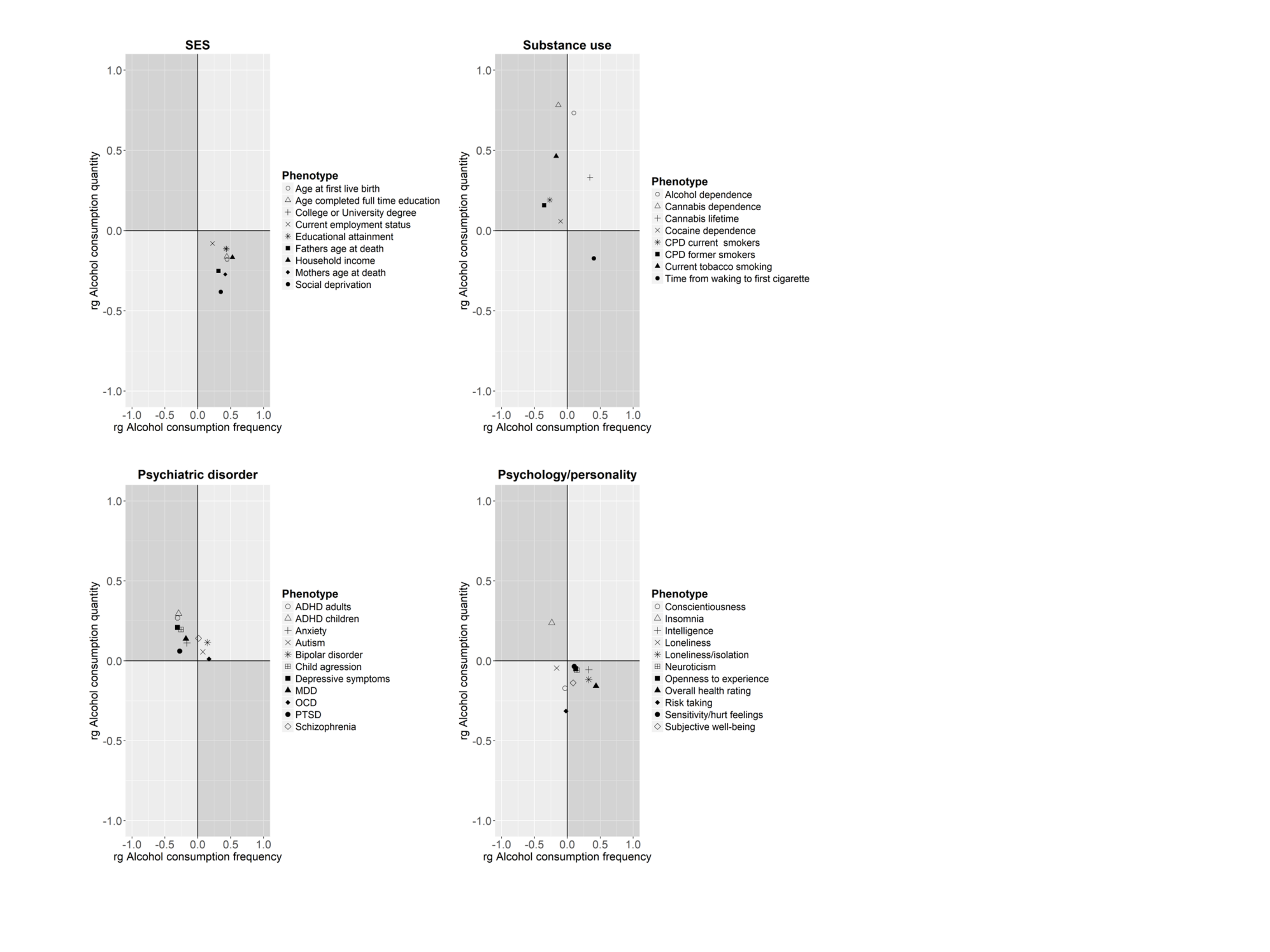
*Supplementary Figure 1. Genetic correlation plots of alcohol consumption frequency and alcohol consumption quantity against traits for each phenotypic category. Dots located in the dark grey quadrants have genetic correlation with opposite directions of effect with alcohol consumption frequency and alcohol consumption quantity, dots in the light grey quadrants have genetic correlations with similar directions of effect.* *Social deprivation scores were reversed so that higher social deprivation/Townsend indicates higher SES.*


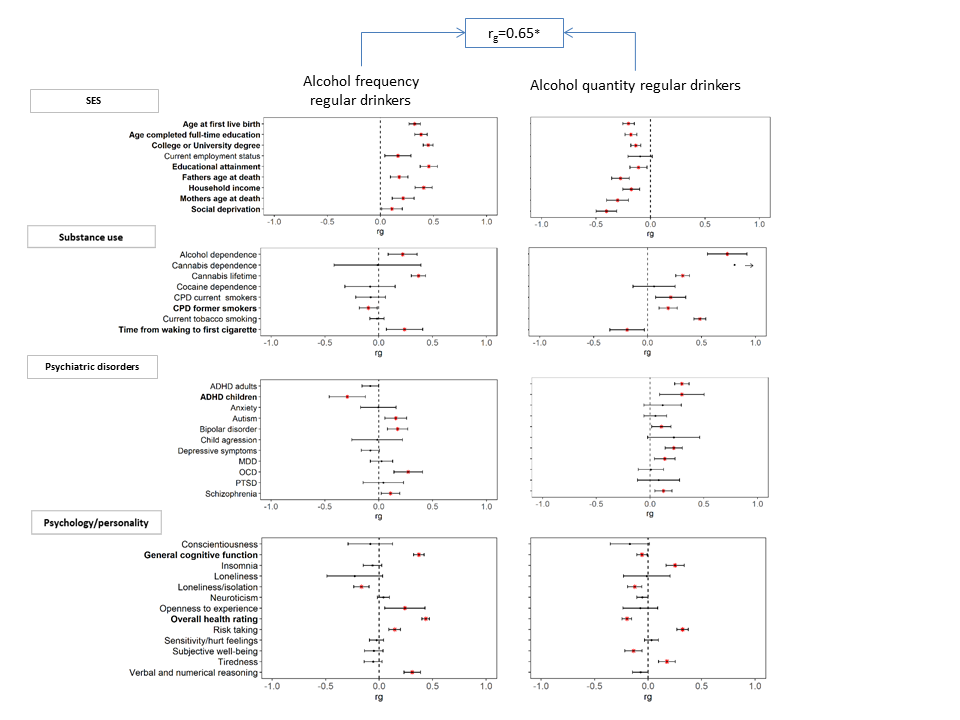


*Supplementary figure 2. Genetic overlap between alcohol frequency (left panels) and alcohol quantity (middle panels) restricted to regular drinkers only against four categories of traits; SES, substance use disorders, psychiatric disorders, and personality/psychological traits. Traits printed in bold show opposite directions of effect. The error bars represent 95% confidence intervals, an arrow towards one indicates a confidence in interval greater than 1 or -1, asterisks indicate significant associations (FDR-adjusted p-value <0.05). Social deprivation scores were reversed so that higher social deprivation/Townsend indicates higher SES.*
